# Scurvy: A Rare Cause of Anemia

**DOI:** 10.7759/cureus.5694

**Published:** 2019-09-18

**Authors:** Frank R Ricaurte, Tariq Kewan, Hamed Daw

**Affiliations:** 1 Westlake High School, Cleveland, USA; 2 Internal Medicine, Cleveland Clinic - Fairview Hospital, Cleveland, USA; 3 Hematology and Oncology, Cleveland Clinic - Fairview Hospital, Cleveland, USA

**Keywords:** scurvy, anemia, vitamin c supplementation, deficiency, perifollicular, hemorrhage

## Abstract

Scurvy is a rather uncommon disease today, and its symptoms can certainly be exhibited with vitamin C deficiency. Dangerously low levels of the vitamin can cause serious health complications and have been proven to be fatal. We present the case of a 42-year-old female with multiple primary diagnoses: easy bruising and Raynaud’s syndrome without gangrene. The purpose of this report is to call attention to the clinical presentation, diagnosis, and treatment of scurvy.

## Introduction

The manifestation of scurvy comes from a lack of vitamin C, indicating that patients with the condition do not consume a sufficient amount of the vitamin in their diets [1]. After one to three months of inadequate vitamin C levels, scurvy leads to a myriad of different symptoms, including anemia, myalgia, bone pain, easy bruising, swelling, hemorrhages, corkscrew hairs, gum disease and gum bleeding, poor wound healing, mood changes, and depression [2]. The disease presents itself in four relatively distinct stages, phases of progression that exhibit increasingly severe symptoms [3]. Early treatment of the disease is essential for regulating the initial symptoms. This primarily entails replenishing vitamin C levels whether it be through a healthier diet of fruits and vegetables or through vitamin C supplements [4].

## Case presentation

**Figure 1:**
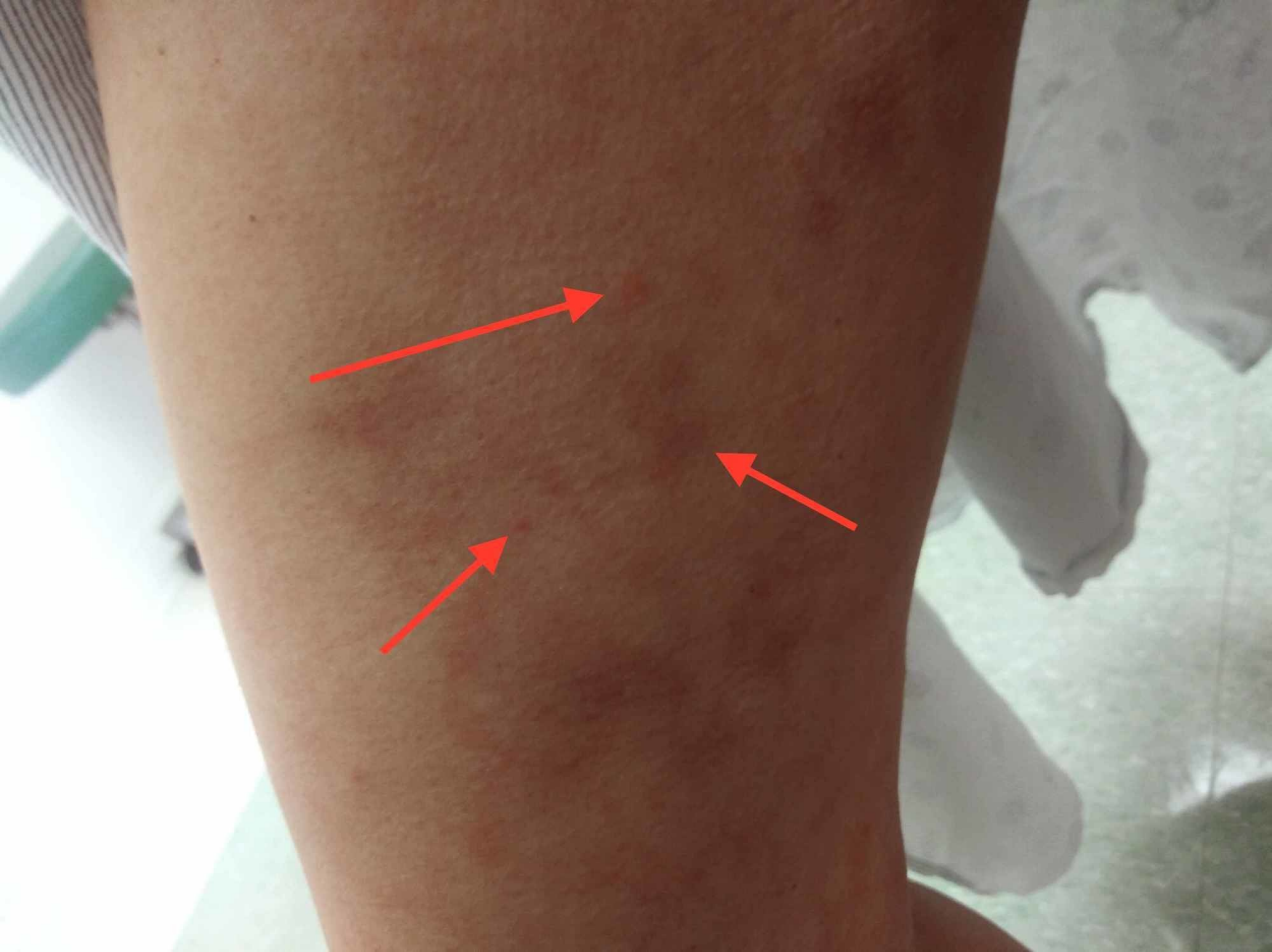
Anterior right arm with petechiae and microhemorrhages around the hair follicles

A 42-year-old female presented with easy bruising and petechiae to her primary care physician and was thus transferred to hematology (Figure 1). Her medical history included chronic obstructive pulmonary disease (COPD), gastroesophageal reflux disease (GERD), human papillomavirus (HPV), mitral valve prolapse (MVP), acute pyelonephritis, anxiety, asthma, essential hypertension, hyperlipidemia, hyperthyroidism, migraines, nephrolithiasis, and obstructive sleep apnea (OSA). The patient has been smoking a half pack of cigarettes daily for the past 10 years. During her office visit, her humoral survey revealed that she had specific antibodies reflecting non-protective titers against the pathogenic bacterium, Haemophilus, and diphtheria-tetanus. The reasons for the said visit included medication follow-up, vitamin level check, urinary urgency, and urinary frequency. These visit overviews reflect the attention given to the patient's symptoms and survey results. 

**Figure 2:**
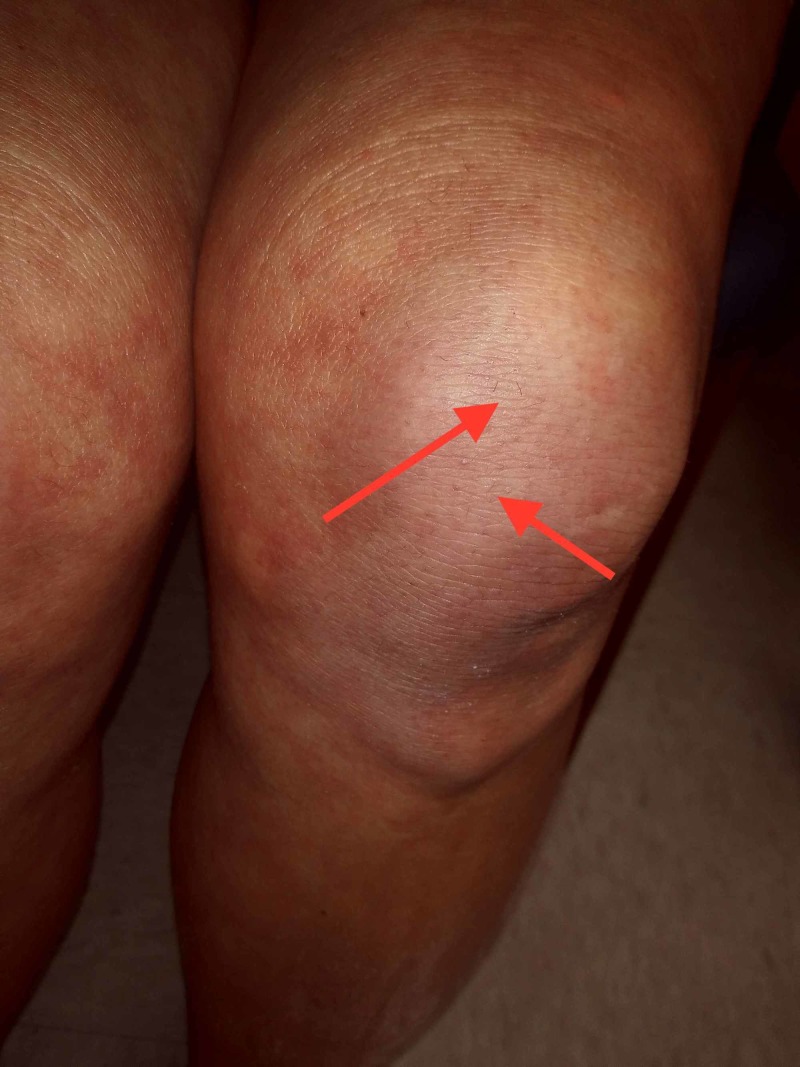
Left lower extremity with anterior perifollicular hemorrhages

The patient had multiple vitamin deficiencies, vitamin C being the most notable of them; her vitamin C count as of December 2018 was 22 umol/L, below what is deemed a healthy amount. However, the patient’s physical examination revealed perifollicular hemorrhage, petechiae, and bruising on various parts of the body (Figure 2).

## Discussion

Scurvy is a disease caused by vitamin C deficiency, a deprivation that today has a prevalence of around 10 to 14% in adults [5]. The disease’s rather low prevalence is quite plausibly a result of the many ways people can obtain adequate amounts of nutrients and vitamins today. Scurvy predominantly presents itself in individuals who abuse drugs and alcohol, live in conditions that prevent proper vitamin nourishment, or have a subpar dietary intake [6]. The overview of water-soluble vitamins written by Pazirandeh and Burns in February 2019 is referenced through the following information [7]. To be more precise regarding what level of vitamin C triggers the manifestation of scurvy’s symptoms, “the plasma concentration of ascorbic acid is most commonly below 0.2 mg/dL under such conditions." Vitamin C has also been allocated to another notable medical purpose: the prevention of cardiovascular disease and cancer. Despite the evident benefits of vitamin C supplementation, there can be several side effects of such treatment, such as causing false-negative stool guaiac results, diarrhea, abdominal bleeding, and increased chances of kidney stones. Hence, vitamin C supplementation is not always widely recommended as an everyday regimen and should only be adhered to in instances where the symptoms of scurvy clearly present themselves.

The patient, in this case, presented with a multitude of symptoms caused by vitamin C deficiency. The initial symptoms of scurvy patients can vary depending on the context of the patient’s situation. Some patients may present with more extreme symptoms, such as hemorrhagic diathesis, although many patients show initial symptoms of anemia, perifollicular hemorrhages, bruising, and pain in upper and lower extremities, as presented in this case [8-9].

Diagnosing a patient with scurvy involves precarious methods of identification. Laboratory tests are not always effective in making a scurvy diagnosis certain. “Plasma ascorbic acid level may help in establishing the diagnosis, but this level tends to reflect the recent dietary intake rather than the actual tissue levels of vitamin C. Signs of scurvy can occur with low-normal serum levels of vitamin C [3]. Recognizing the history of patients’ vitamin C deficiency in regards to their dietary intake and, more specifically, their tissue level data leads to a more accurate diagnosis of scurvy.

Unlike scurvy’s past treatment efforts that were often futile as per the disease’s extremely high mortality rate, scurvy today is surely treatable. The current treatment is rather straightforward as, in most cases, it consists of standard vitamin C supplementation. The amount given to patients typically decreases within a few weeks of the original given dosage [2, 10]. The starting amount obviously depends on the severity of the disease at the time of the diagnosis, but the gradual decrease in dosage over time is proven to steadily lessen symptoms of the disease. Identifying the disease, which primarily is accomplished by means of recognizing petechiae, perifollicular hemorrhage, and bruising, is most influential in the primary dosage given to patients; severity increases over the time during which the deficiency is sustained. After a few months of supplementation, patients’ vitamin levels are typically in accordance with the recommended dietary allowance (RDA). Commonly targeted in many scurvy cases, vitamin C therapy is used to replete the body with a proper dietary plan and vitamin C supplements [8-9].

## Conclusions

This case emphasizes the recognition required to identify scurvy and the potential consequences that would result if its diagnosis was otherwise not made. Some symptoms of the presented patient, namely petechiae, were certainly more subtle than the more extreme instances of scurvy's symptoms in other cases. Nevertheless, this case's presentation of scurvy and its subsequent remission still remain valuable in aiding the proper handling of the disease. The treatment of scurvy discussed should be used with reference to the relativity of each patient’s severity of symptoms, along with the disease’s progression.
